# Enhancing the Physiochemical Properties of Puerarin via L-Proline Co-Crystallization: Synthesis, Characterization, and Dissolution Studies of Two Phases of Pharmaceutical Co-Crystals

**DOI:** 10.3390/ijms22020928

**Published:** 2021-01-18

**Authors:** Muhammad Inam, Lu Liu, Jian-Wei Wang, Ka-Xi Yu, Chi-Uyen Phan, Jie Shen, Wen-Hua Zhang, Guping Tang, Xiurong Hu

**Affiliations:** 1 Department of Chemistry, Zhejiang University, Hangzhou 310028, China; Inam_amandi90@yahoo.com (M.I.); lulu0698@126.com (L.L.); wjw126hz@zju.edu.cn (J.-W.W.); yukaxi@zju.edu.cn (K.-X.Y.); pcuyen@ute.udn.vn (C.-U.P.); shenjie1003@zju.edu.cn (J.S.); tangguping@zju.edu.cn (G.T.); 2 College of Chemistry, Chemical Engineering and Materials Science, Soochow University, Suzhou 215123, China; whzhang@suda.edu.cn

**Keywords:** puerarin, L-proline, pharmaceutical co-crystal, crystal structure, solubility and dissolution rate, Chinese traditional medicine, Hirschfeld analysis, hydrogen-bonding

## Abstract

Puerarin (PUE) is a Chinese traditional medicine known to enhance glucose uptake into the insulin cells to downregulate the blood glucose levels in the treatment of type II diabetes. Nevertheless, the bioavailability of pristine PUE is limited due to its poor solubility and low intestinal permeability. In this work, we demonstrate that the solubility of PUE can be significantly enhanced via its co-crystallization with L-Proline (PRO). Two crystalline phases, namely, the solvate-free form [PUE][PRO] (**I**) and the solvated form [PUE]_2_[PRO]∙EtOH∙(H_2_O)_2_ (**II**) are isolated. These two phases are characterized by single-crystal X-ray diffraction (SCXRD), powder X-ray diffraction (PXRD), Fourier-transformed infrared (FT-IR) spectra, nuclear magnetic resonance (NMR), and thermogravimetric analysis in association with differential scanning calorimetry (TGA-DSC). The solubility and dissolution rate of both **I** and **II** in water, gastrointestinal tract at pH 1.2, and phosphate buffer at pH 6.8 indicates a nearly doubled increase as compared to the pristine PUE. 3-(4,5-Dimethylthiazol-2-yl)-2,5-diphenyltetrazolium bromide (MTT) assay of pristine PUE, **I** and **II** against murine colon cancer cell lines CT-26 and human kidney cell lines HEK-293 indicated that neither compound exhibits obvious cytotoxicity after 24 h. This work showcases that the readily available and biocompatible PRO can be a promising adjuvant to enhance the physicochemical properties of PUE toward orally administered drug formulation with improved pharmacokinetics.

## 1. Introduction

One of the primary goals in pharmaceutical crystallization is to increase the solubility of the otherwise less soluble drugs to improve the bioavailability of the active pharmaceutical ingredients (APIs) toward the better pharmacokinetic outcome [1,2,3]. The core of such endeavor underlies the exploitation of different forms of APIs, such as co-crystals, salts, solvates (including hydrates), and polymorphs, followed by their physicochemical properties optimization [4]. Among these forms, co-crystallization is widely used to optimize the physicochemical properties of APIs due to the retention of its intrinsic pharmaceutical properties [5,6,7,8,9,10,11,12]. The pharmacokinetic properties can be regulated through shaping the co-crystals to improve its solubility and dissolution rate [13,14,15,16], mechanical stability [17], hygroscopicity [18], and ultimately bioavailability [10].

**Scheme 1:**
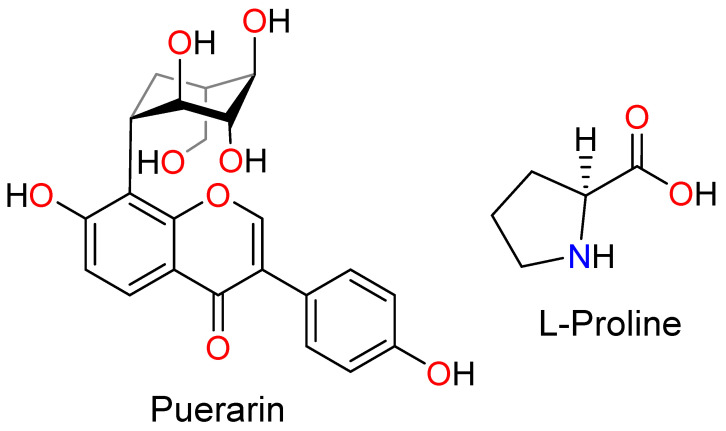
The structure of Puerarin and l-Proline.

Puerarin (PUE, Scheme 1) is a major active ingredient in Chinese traditional medicine (CTM) that originates from the kudzu root known as Gegen [19]. PUE has been utilized for the treatment of diabetes mellitus in China since the 1990s to enhance the glucose uptake into the insulin cell, and subsequently downregulate the blood glucose levels in the type II diabetes treatment [20]. Notably, PUE has also been reported to inhibit the proliferation of a variety of cancer cells, such as colorectal cancer [21,22,23], colon cancer [23,24,25], and breast cancer [26]. However, because of its sizable conjugated skeleton, PUE exhibits poor solubility, leading to low oral bioavailability [27]. According to the biopharmaceutics classification system (BCS), PUE can be categorized as IV class due to its low solubility and limited intestinal permeability [28]. Besides, PUE also exhibits a short elimination half-life which further hindered its clinical adaption [29]. To improve the oral bioavailability and circulation time of PUE, a paucity of methodologies have been developed, such as the PUE encapsulation by HP-β-CD-PLGA nanoparticles [30], lipid nanoparticles [31], as well as RGD-appended PEGylated lipid nanoparticles [29].

In this work, we demonstrate that the solubility of PUE can be readily improved via its co-crystallization with L-proline (PRO, Scheme 1). PRO is well-known to exhibit good potential for co-crystal formation due to the presence of both hydrogen-bonding donor and acceptor sites in the molecule [32,33,34]. Two different phases of PUE-PRO co-crystals, namely the solvate-free form [PUE][PRO] (**I**) and the solvated form [PUE]_2_[PRO]∙EtOH∙(H_2_O)_2_ (**II**), have been prepared under different crystallization conditions and characterized spectroscopically. Compounds **I** and **II** represent the first cases of PUE-based co-crystals. As compared to those of the pristine PUE, the solubility and dissolution rates of **I** and **II** in water (pH 7.0), gastrointestinal tract at pH 1.2, and phosphate buffer at pH 6.8 indicated a nearly doubled increase. 3-(4,5-Dimethylthiazol-2-yl)-2,5-diphenyltetrazolium bromide (MTT) assay of pristine PUE, **I** and **II** against murine colon cancer cell lines CT-26 and human kidney cell lines HEK-293 indicated that neither compound exhibits obvious cytotoxicity upon culturing the cells for an extended time of 24 h and 48 h, respectively. This work demonstrates that PUE co-crystal forms of **I** and **II** with the presence of readily available and biocompatible PRO can be a promising alternative to pristine PUE with better pharmacokinetic outcomes.

## 2. Results and Discussion

### 2.1. Synthesis and Co-Crystal Characterizations

The preferred route for the uptake of drugs, including Chinese traditional medicine (CTM), is the oral administration because of its convenience, low cost, and high persevering acquiescence compared to other routes. However, many of the bioactive ingredients of CTM are poorly soluble, leading to low oral bioavailability and delivery problems. Specifically for PUE, the oral bioavailability is only about 7% [32,33,34,35], and the dominant drug uptake at present is through intravenous injection. Thus, in the current study, the co-crystallization method using L-proline (PRO) as the partner excipient is employed to improve the water solubility of PUE. PRO was employed as a model excipient due to its good biocompatibility, its presence of both donor and acceptor sites for hydrogen-bonding, and its good solubility in water to guarantee the overall solubility of the product [36,37].

**Figure 1:**
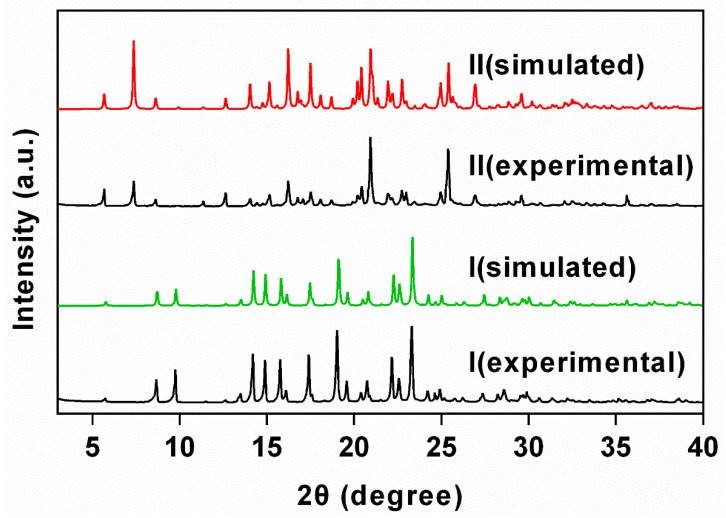
PXRD patterns of **I** and **II** showing a good agreement between the experimental and those simulated from single-crystal diffraction data and thus high phase purity of **I** and **II**.

Both **I** and **II** were prepared as single crystals from equivalent molar of PUE and PRO in EtOH/H_2_O (9:1, *v*/*v*) under different precipitation temperatures. In the ^1^H nuclear magnetic resonance (NMR) spectra of **I** and **II**, peaks in the down-field area (δ 6.79–8.35 ppm) and the up-field area (δ 1.65–2.07 ppm) are diagnostic of the presence of PUE and PRO (Supplementary Figure S1). The triplet at δ 1.05 ppm in **II** further indicates the inclusion of EtOH solvate within the crystalline sample. The experimental powder X-ray diffraction (PXRD) patterns of **I** and **II** agree well with those simulated from single-crystal X-ray diffraction data (Figure 1), indicating highly phase purity of both complexes. In the Fourier-transform infrared (FT-IR) spectra (Supplementary Figure S2), peaks at 1605/1419 cm^−1^ (**I**) and 1590/1398 cm^−1^ (**II**) were assignable as the asymmetric/symmetric vibrations of COO^−^ in PRO [38,39,40]. Notably, the FT-IR spectra for both **I** and **II** feature no peak at around 1700 cm^−1^, indicating that the carboxylate remains deprotonated and the proton migrates to the –NH– to give a quaternary amine–carboxylate zwitterionic pair [41], as typically found in proline-based compounds [42,43].

The thermogravimetric analysis coupled with differential scanning calorimetry (TGA-DSC) reveals that **I** exhibit no obvious solvate loss until 224 °C (endothermic), followed by dramatic decomposition (Supplementary Figure S3a). This strongly suggests that **I** crystallizes as a solvate-free form. Meanwhile, the TGA-DSC curves of **II** show smooth and continuous solvate loss before 150 °C (Supplementary Figure S3b). This is followed by a plateau until 211 °C, at which point the PUE and PRO pair start to decompose. The total weight loss of 5.8% before 211 °C corresponds to the loss of two aqua and one EtOH molecules (calculated 7.8%), and the slight deviation might due to part of the solvate loss (mainly EtOH) during sample preparation.

### 2.2. Crystal Structure Analysis

**Figure 2:**
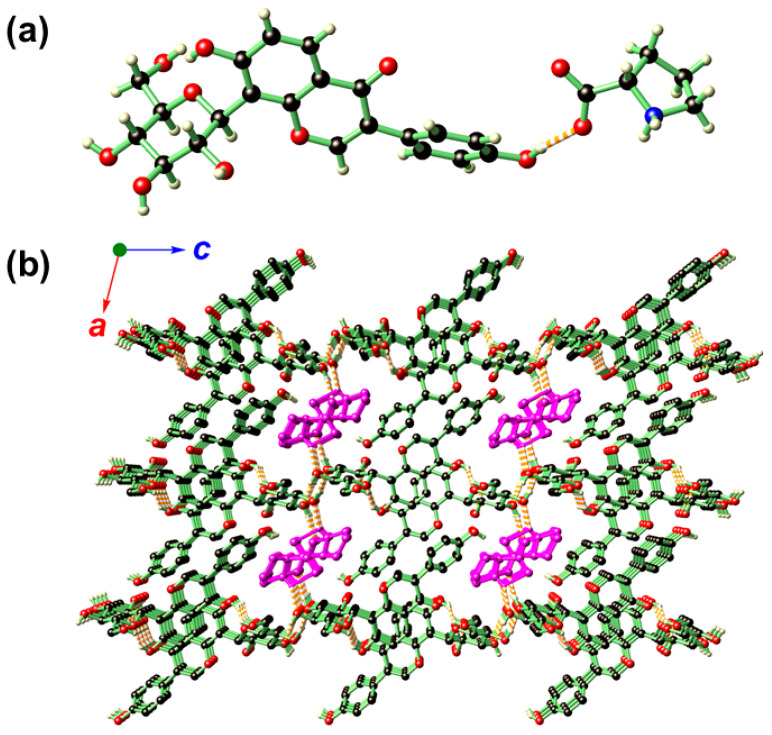
The asymmetric unit (**a**) and hydrogen-bonding network (**b**) of **I**. In b, the structure is propagated along the crystallographic *a* and *c* directions showing that PRO (distinguished by dark magenta for clarity purpose) resides inside the 1D channels generated by stacking of the PUE molecules. Color codes: O (red), N (blue), C (black), H (light yellow).

Phase **I** crystallizes in the monoclinic space group *P*2_1_ (Table 1) with its asymmetric unit featuring one PUE and one PRO molecules (Figure 2a). In the PRO moiety of **I**, the two C–O distances are nearly identical (1.249(3) Å and 1.255(3) Å), indicating that the carboxylate is in a conjugated and uncoordinated fashion, and the proton on the –COOH migrates to the –NH to give a zwitterion, corroborating that observed in the FT-IR spectrum. Such an averaged C–O distances are similar to those zwitterions based on metal-carboxylate pairs, such as *trans*-[Zn(*iso*-hmnH)_2_(H_2_O)_2_] and *cis*-[Zn(hmnH)_2_(H_2_O)_2_]·2H_2_O [44,45], and hemi-zwitterions such as [CuCl(*iso*-hmnH_2_)(*iso*-hmnH)]·H_2_O and [CuCl(hmnH_2_)(hmnH)] (*iso*-hmnH = 2-(hydroxymethyl) isonicotinate; hmnH = 2-(hydroxymethyl)nicotinate) [46].

Compound **I** is rich hydrogen-bonding interactions (Supplementary Table S1), and as a result, the PUE molecules are arranged to give a layered structure within the crystallographic *ab* plane (Figure 2b), and these layers are further stacked along the *c* direction in such a way that one-dimensional channels along the *b* direction is generated to accommodate PRO molecules.

**Figure 3:**
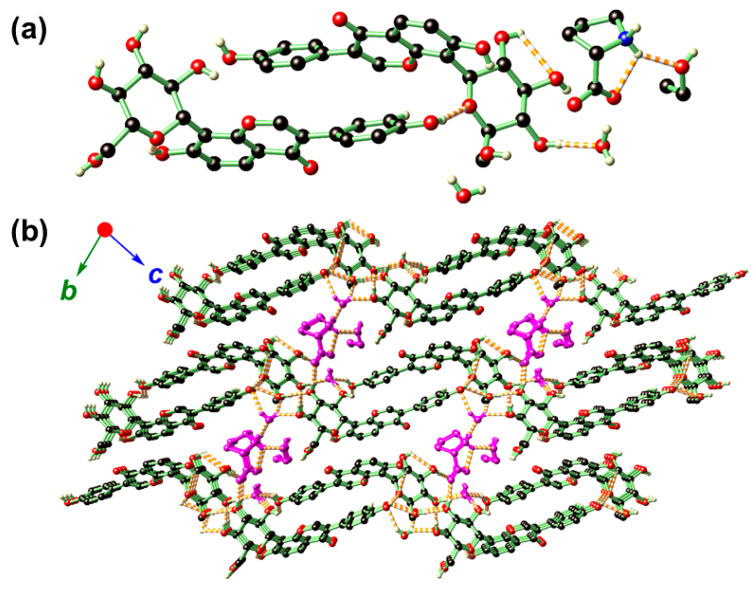
The asymmetric unit (**a**) and hydrogen-bonding network (**b**) of **II**. In b, the structure is propagated along the crystallographic *b* and *c* directions showing that PRO, one EtOH, and a pair of H_2_O molecules are sandwiched in-between the layers generated by PUE. The PRO and crystalline solvates were distinguished by dark magenta for clarity purposes. Color codes: O (red), N (blue), C (black), H (light yellow).

Crystal structure analysis revealed that the solvated form of **II** belongs to the triclinic *P*1 space group (Table 1), and its asymmetric unit consists of two PUE, one PRO, one EtOH, and two aqua molecules (Figure 3a). In **II**, the PRO moiety also remains in its zwitterionic form similar to that identified in **I**. The two PUE molecules are in a head-to-tail arrangement as supported by hydrogen-bonding. With the additional presence of one PUE, and crystalline solvates EtOH and H_2_O, the hydrogen-bonding interactions in **II** are more complex than that of **I** (Supplementary Table S1). Similar to that found in **I**, PUE is also found to stack within the crystallographic *ab* plane (Figure 3b). Nevertheless, the presence of EtOH and aqua solvates marginally increased the separations between these layers to finally yield a sandwich-type structure in which the PRO and its hydrogen-bonded solvates reside in-between these layers. It is notable that EtOH is associated with PRO via a single hydrogen bond and can be readily extruded from the network without disturbing the overall structural integrity of **II**. This may serve to explain the slight deviation of experimental weight loss (5.8%) from that of the calculated (7.8%) observed in the TGA-DSC diagram (Supplementary Figure S3b).

### 2.3. Hirshfeld Surface Analysis of TAF, **I** and **II**

**Figure 4:**
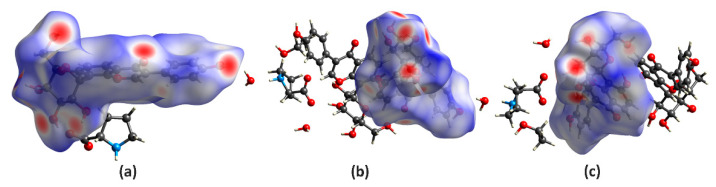
Hirshfeld surfaces for the PUE with its neighboring molecules in the crystals of **I** (**a**), **II-A** (**b**), and **II-B** (**c**).

Hirshfeld surface (HS) analysis serves as a powerful tool to figure out the packing modes and intermolecular interactions in molecular crystals [47]. To evaluate and compare the nature of close interactions between the different lattice components that contributed to the supramolecular assembly of **I** and **II**, the three-dimensional (3D) HS and the two-dimensional (2D) fingerprint maps were generated using the Crystal Explorer program [48]. As illustrated in Figure 4, the deep-red spots on the surface portrait the shortest N–H∙∙∙N, N–H∙∙∙O, O–H∙∙∙N, and O–H∙∙∙O interactions, while other visible spots on the surfaces represent subtle H∙∙∙H contacts. Overall, the HS of **I** and **II** differ from each other in shape, reflecting the different weights of intermolecular contacts.

**Figure 5:**
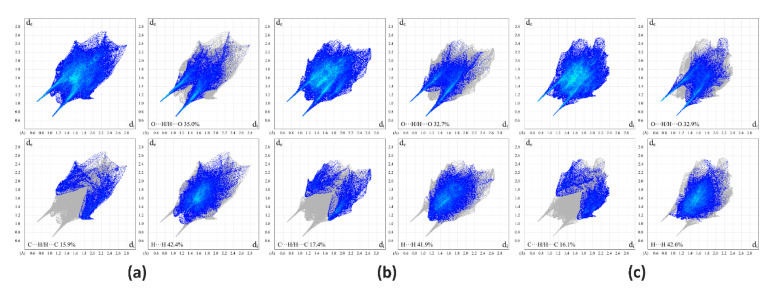
Two-dimensional fingerprint plots of **I** (**a**), **II**-A (**b**), and **II**-B (**c**), showing the contribution of each type of contact within the molecule.

**Figure 6:**
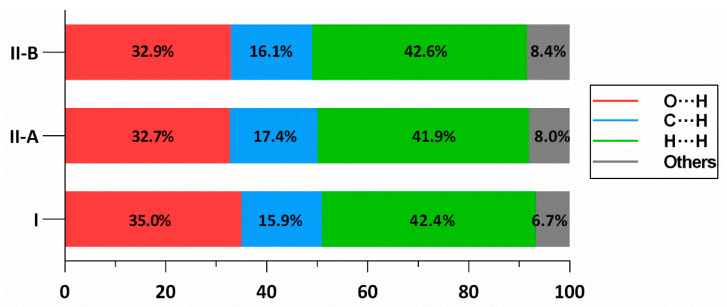
Relative contribution ratios of each type of contacts within the structure of **I**, and two parts of **II** (denoted as **II**-A and **II**-B), showing that the presence of water molecules in **II** raises the collective contribution of O∙∙∙H/H∙∙∙O amongst these interactions.

A summary of intermolecular contacts for PUE and PRO are illustrated with decomposed fingerprint plots (Figure 5) to showcase detailed atom-pair close contacts, including H∙∙∙H, O∙∙∙H/H∙∙∙O, and C∙∙∙H/H∙∙∙C contacts. Intermolecular interactions appear as discrete spikes in the 2D fingerprint plots. The quantitative analysis (Figure 6) shows that the H∙∙∙H and O∙∙∙H/H∙∙∙O interactions are the largest contributors to the total HS for **I** and **II** as compared to C∙∙∙H/H∙∙∙C and other contacts, represented by the large surfaces (blue) in the fingerprint plots.

### 2.4. Solubility and Dissolution Rates of PUE, **I** and **II**

It is well-established that the solid form of APIs has extensive influence on the solubility and dissolution rate of the drug. Consequently, it is vital to choose a suitable API solid form for successful drug expansion. According to the biopharmaceutics classification system (BCS) system, PUE is categorized as a class IV drug, meaning low solubility and low intestinal permeability. The supersaturation solid form of API may increase the bioavailability and permeability.

**Figure 7:**
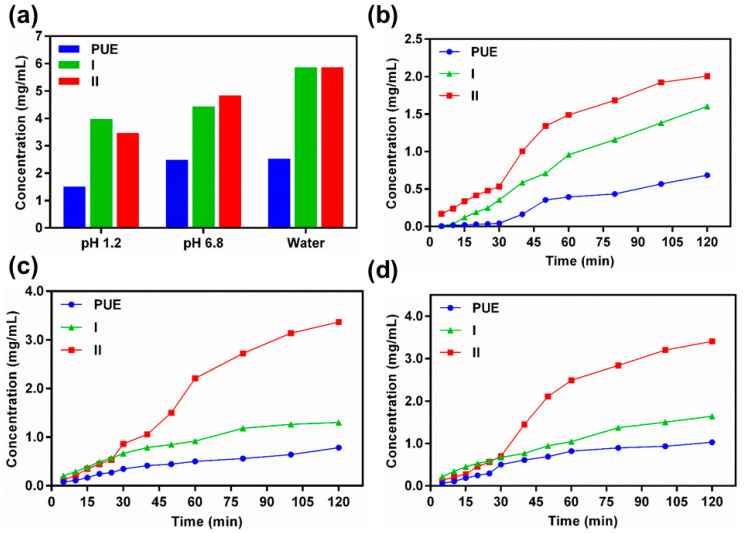
A summary of the solubility profile of PUE, **I** and **II** in the gastrointestinal tract at pH 1.2, phosphate buffer at pH 6.8, and H_2_O at pH 7.0 (**a**), as well as the time-dependent dissolution profile of these compounds under respective conditions: gastrointestinal tract at pH 1.2 (**b**), phosphate buffer at pH 6.8 (**c**), and H_2_O at pH 7.0 (**d**), reflecting the dissolution rate of each compound. Data are expressed as average values from three replicates.

The comprehensive solubility of PUE, **I**, and **II** in the gastrointestinal tract at pH 1.2, phosphate buffer at pH 6.8, and H_2_O at pH 7.0 are depicted in Figure 7 as well as in Tables S2 and S3 (Supplementary Material). Both **I** and **II** exhibit nearly doubled solubility as compared to PUE with the solvated-free form **I** been comparable to the solvated form of **II**. In contrast, **II** also exhibits higher dissolution rate than both **I** and PUE. The exceptional solubility of **I** and **II**, and dissolution rate of **II** as compared to **I** and PUE might due to the presence of rich hydrogen-bonding interactions that enables simultaneously more competitive interactions with the solution media to facilitate the dissociation of the subcomponents of the co-crystal. Besides, the sandwich-type structure of **II** with solvate-accessible layer separations (Figure 3b) as compared to the channel structure of **I** may also result in the quick entrance of solution media into the crystal lattice to accelerate the dissolution of **II**. Thus, the increase of dissolution can be observed in the order **II** > **I** > PUE, and the solubility and dissolution of PUE were successfully improved via co-crystallization with PRO.

### 2.5. MTT Assays

**Figure 8:**
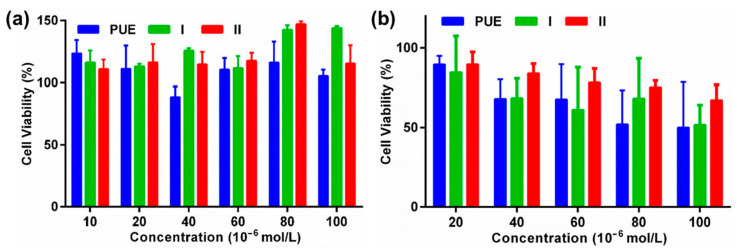
The cell viability data of PUE, **I**, and **II** against murine colon cancer CT-26 (**a**) and human kidney cell lines HEK-293 (**b**) upon culturing 24 h.

PUE is widely reported to inhibit the proliferation of various cancer cells such as colon cancer [23,24,25]. It is reported that PUE is capable of inducing the apoptosis of colon cancer HT-29 cells [49]. To further evaluate the potential of PUE, **I**, and **II** as anti-cancer agents, we evaluated the biological activities of the pristine PUE, **I**, and **II** against the proliferation of CT-26 cancer cell lines (murine origin) and HEK-293 (human origin) cell lines using the 3-(4,5-dimethylthiazol-2-yl)-2,5-diphenyltetrazolium bromide (MTT) assay. As shown in Figure 8, PUE, **I**, and **II** exhibit negligible cytotoxicity toward CT-26 and HEK-293 cell lines upon culturing the cells with the drugs for 24 h. These complexes are also comparatively more toxic toward HEK-293 than CT-26 cell lines, and the CT-26 cells were also well survived upon incubating for additional 24 h (Supplementary Figure S4).

## 3. Materials and Methods

### 3.1. General

Puerarin (PUE) and L-Proline (PRO) were respectively purchased from J&K Scientific Ltd (Beijing, China) and Aladdin (Shanghai, China). Roswell Park Memorial Institute (RPMI) 1640 cell culture medium (for CT-26) and Dulbecco’s modified Eagle medium (DMEM) for HEK-293 were purchased from Bristol-Myers Squibb Trading Co. Ltd (New York, NY, USA). 3-(4,5-Dimethylthiazol-2-yl)-2,5-diphenyl tetrazolium bromide (MTT) was obtained from Sigma (St. Louis, MO, USA). All the other chemicals were obtained directly from commercial sources and used as received. The ^1^H nuclear magnetic resonance (NMR) spectra were recorded on a Bruker DRX-400 spectrometer (Bruker, Ettlingen, Germany) and chemical shifts (δ) are referenced to the residual solvent peaks or internal standard TMS. FT-IR spectra were measured on a Thermo Scientific Nicolet iS50 FT-IR spectrometer (Thermo Fisher Scientific Co., Waltham, MA, USA) as KBr disks (400–4000 cm^−1^). Powder X-ray diffraction (PXRD) patterns were recorded on a Rigaku D/Max-2550PC micro-diffractometer (Rigaku Corporation, Tokyo, Japan). A rotating-anode Cu-target X-ray (λ= 1.5406 Å) was used, which was worked at 40 kV, 250 mA with the scanning ranges of 3.0 to 40.0° and scanning speed of 5° min^−1^ with an increasing step size of 0.02° and count time of 0.5–2 s. The TGA-DSC investigation was performed on a TA DSC Q100 differential filtering calorimeter (TA Instruments, New Castle, DE, USA) at the heating rate of 10 °C min^−1^ under a nitrogen stream of 50 cm^3^ min^−1^. Ultraviolet-visible (UV–vis) spectra were collected on a Thermo Scientific Evolution 300 UV spectrometer (Thermo Scientific, Waltham, MA, USA). The cytotoxicity data were evaluated on a Bio-Rad Elisa Plate Reader 680 (Bio-Rad, Hercules, CA, USA).

### 3.2. Synthesis of Co-Crystals **I** and **II**

The co-crystal was synthesized via the recrystallization method [50]. PUE (0.832 g, 2.0 mmol) and PRO (0.230 g, 2.0 mmol) were dissolved in 0.6 mL of EtOH (90%) and the solution was stirred at 90 °C for 30 min and then cooled to 50°C. The plate-shaped colorless crystals of **I** were formed after 24 h. The same amount of PUE and PRO were stirred at 50°C followed by cooling to room temperature yielded colorless fine needle crystals of **II** after two days.

*Characterization Data for **I**.*^1^H NMR (400 MHz, DMSO-*d*_6_) δ 9.60 (br, 1H), 8.35 (s, 1H), 7.93 (d, *J* = 8.8 Hz, 1H), 7.39 (d, *J* = 8.7 Hz, 2H), 7.01 (d, *J* = 8.8 Hz, 1H), 6.80 (d, *J* = 8.6 Hz, 2H), 5.01 (br, 2H), 4.80 (d, *J* = 9.8 Hz, 2H), 4.54 (br, 1H), 4.03 (t, *J* = 9.2 Hz, 1H), 3.63–3.73 (m, 2H), 3.18–3.43 (m, 6H), 2.97–3.04 (m, 1H), 1.98–2.05 (m, 1H), 1.85–1.98 (m, 1H), 1.75–1.82 (m, 1H), 1.65–1.74 (m, 1H). IR (KBr pellet): 3398 (br), 3248 (sh), 2923 (m), 1632 (s), 1592 (s), 1515 (s), 1446 (s), 1398 (s), 1268 (s), 1214 (s), 1176 (m), 1085 (s), 1061 (s), 1014 (s), 890 (m), 837 (m), 797 (m), 632 (m), 602 (m), 546 (m) cm^−1^.

*Characterization Data for **II**.*^1^H NMR (400 MHz, DMSO-*d*_6_) δ 9.56 (br, 3H), 8.35 (s, 2H), 7.93 (d, *J* = 8.8 Hz, 1H), 7.39 (d, *J* = 8.5 Hz, 2H), 7.06–6.91 (m, 1H), 6.80 (d, *J* = 8.6 Hz, 2H), 5.00 (br, 4H), 4.81 (br, 4H), 4.53 (br, 2H), 4.37 (br, 2H), 4.02 (br, 2H), 3.61–3.73 (m, 4H), 3.41–3.45 (m, 5H), 3.18–3.28 (m, 8H), 3.00–3.03 (m, 2H), 1.89–2.04 (m, 3H), 1.66–1.79 (m, 3H), 1.05 (t, *J* = 7.0 Hz, 3H). IR (KBr pellet): 3361 (br), 3266 (sh), 3084 (m), 3018 (m), 2924 (w), 2855 (w), 2736 (w), 2673 (w), 2607 (w), 1646 (s), 1605 (s), 1515 (s), 1445 (s), 1387 (s), 1358 (s), 1339 (s), 1258 (s), 1280 (s), 1180 (s), 1085 (s), 1043 (s), 1015 (m), 989 (s), 943 (s), 888 (w), 834 (m), 795 (w), 773 (w), 737 (w), 657 (w), 630 (m), 545 (m), 514 (m), 490 (w), 440 (w) cm^−1^.

### 3.3. Single-Crystal X-ray Crystallography

Single-crystal X-ray diffraction data were collected on a Bruker SMART APEX II diffractometer (Bruker, Ettlingen, Germany) using graphite-monochromated Cu Kα radiation (λ = 1.54178 Å). The collected data were corrected for absorption with SADABS [51]. The structures were solved by direct methods and refined by full-matrix least-squares on *F*^2^ using SHELXTL-2016 [52]. Non-hydrogen atoms were refined anisotropically. The H atoms on the phenolic and alcoholic –OH, water, and cationic NH_2_ were placed in calculated positions either by HFIX instructions or by Calc-OH program in the WinGX suite [53]. Crystallographic data for the crystal structures have been deposited in the Cambridge Crystallographic Data Centre (CCDC) with supplementary numbers of 2041676 (**I**) and 2041677 (**II**). These data can be obtained free of charge from the CCDC via www.ccdc.cam.ac.uk/data_request/cif. A summary of the key crystallographic data are listed in Table 1.

### 3.4. Solubility and Dissolution Measurement

The solubility and dissolution studies of pure PUE, **I**, and **II** were measured using a Thermo Scientific Evolution 300 UV spectrometer in deionized water at pH 7.0, gastrointestinal tract at pH 1.2, and phosphate buffer at pH 6.8 solution at 37 °C. The concentration was calculated utilizing a standard curve, which was made by measuring the absorbance of different concentrations at their λ_max_. A superfluity sum of the sample was added to 10 mL of H_2_O medium, pH 1.2 and pH 6.8 phosphate, respectively.

The oversaturated solution was stirred at 500 RPM by magnetic stirring at 37 °C for 24 h and the suspension was filtered through Whatman’s 0.45 mm syringe channel, diluted sufficiently and the absorbance was measured at their λ_max_. For the dissolution rate, the sample was specifically poured into 250 mL pH 7.0 water, pH 1.2 gastrointestinal tract, and pH 6.8 phosphates medium and then stirred at 150 RPM for 2h at 37 °C. Each solution was measured by the absorbance at λ_max_. At customary interims, the solution was replenished with 3 mL of the respective blank solution to maintain an overall steady volume.

### 3.5. Cytotoxicity Evaluation by MTT Assay

CT-26 and HEK-293 cell line was purchased from American Type Culture Collection (Manassas, VA, USA). The cell lines were cultured in RPMI 1640 (for CT-26) or DMEM (for HEK-293) containing 10% fetal bovine serum (FBS, Zhejiang Tianhang Biotechnology Co. Ltd., Hangzhou, China) and 1% penicillin/streptomycin (Zhejiang Tianhang Biotechnology Co. Ltd., Hangzhou, China). Cells grew as a monolayer and were detached upon confluence using trypsin (0.5% *w/v* in PBS, Zhejiang Tianhang Biotechnology Co. Ltd., Hangzhou, China). The cells were harvested from the cell culture medium by incubating in trypsin solution for 3 min. The cells were centrifuged, and the supernatant discarded. A 3 mL portion of serum-supplemented cell culture medium was added to neutralize any residual trypsin. The cells were re-suspended in serum-supplemented RPMI 1640 (for CT-26) or DMEM (for HEK-293) at a concentration of 5 × 10^4^ cells per 1 mL. Cells were cultured at 37 °C and 5% CO_2_ for MTT studies.

The CT-26 or HEK-293 cells were seeded at a density of 1 × 10^4^ cells per well in 100 µL of complete RPMI 1640 (CT-26) or DMEM (for HEK-293), and cultured for 16 h for attachment. The culture medium was then replaced by a serum-free medium containing various concentrations of pristine PUE, **I**, and **II**. After incubation with a period of 24 h and 48 h, the MTT solution (100 μL, 0.5 mg/mL in serum-free RPMI 1640 medium for CT-26 and DMEM for HEK-293) was added to replace the cell culture medium. After incubating the cells at 37 °C for 4 h, the MTT solution was removed and DMSO (100 μL) added to dissolve the formazan crystals formed, and the microplates were agitated for 5 min at a medium rate before the spectrophotometric measurement at a wavelength of 570 nm on a microplate reader. The untreated cells served as the 100% cell viability control, while the completely dead cells served as the blank. All experiments were carried out with four replicates. The relative cell viability (%) related to control cells was calculated by the formula below:

$$V\%=\frac{{\left[A\right]}_{experimental}-{\left[A\right]}_{blank}}{{\left[A\right]}_{control}-{\left[A\right]}_{blank}}\times 100\%$$

where V% is the percentage of cell viability, [*A*]*_experimental_* is the absorbance of the wells culturing the treated cells, [*A*]*_blank_* is the absorbance of the blank, and [*A*]*_control_* is the absorbance of the wells culturing untreated cells.

## 4. Conclusions

Two new forms of PUE have been successfully obtained and structurally authenticated via its co-crystallization with abundant and naturally-occurring PRO. The solubility and dissolution data suggested that the obtained co-crystal forms **I** and **II** exhibit marginal enhancement as compared to the pristine PUE. The solvated form of **II** further outperforms that of **I** probably due to its rich hydrogen-bonding interactions and sandwich-type structure that facilitate the quick diffusion of solution media. The use of the bio-compatible and non-toxic natural product as a co-delivery reagent may point to new directions in pharmaceutical co-crystallization to produce drug formulations with synergistic functions.

## Figures and Tables

**Table 1:** Summary of crystallographic data for **I** and **II**.

Compound	I	II
Empirical formula	C_21_H_20_O_9_∙C_5_H_9_NO_2_	2(C_21_H_20_O_9_)·C_5_H_9_NO_2_·2H_2_O·C_2_H_5_OH
Formula Weight	531.50	1029.97
Crystal system	monoclinic	Triclinic
Space group	*P*2_1_	*P*1
*a*/Å	10.2463 (4)	6.3123 (2)
*b*/Å	7.8656 (3)	12.1389 (4)
*c*/Å	15.4520 (6)	15.7274 (6)
*α*/°	90	81.7330 (10)
*β*/°	97.7830 (10)	89.7330 (10)
*γ*/°	90	87.4870 (10)
*V*/Å^3^	1233.86 (8)	1191.43 (7)
*Z*	2	1
*D_c_*/(g cm^−3^)	1.431	1.436
*μ* (Mo-Kα)/mm^−1^	0.952	0.974
*F*(000)	560	544
Total reflections	13,357	40,998
Unique reflections	4414	9385
No observations	4411	9322
No parameters	349	673
Flack parameter	0.03 (3)	−0.01 (3)
*R*_int_	0.0254	0.0293
*R ^a^*	0.0293	0.0332
*wR ^b^*	0.0774	0.0957
*GOF ^c^*	1.057	1.080

*^a^ R*_1_ = Σ||F_o_| − |F_c_||/Σ|F_o_|, *^b^* wR_2_ = {Σ[ω(F_o_^2^ − F_c_^2^)^2^]/Σ[ω(F_o_^2^)^2^]}^1/2^, and *^c^* GOF = {Σ[ω(F_o_^2^ − F_c_^2^)^2^]/(n − p)}^1/2^, where *n* is the number of reflections, and *p* the total number of parameters refined.

## Data Availability

The data presented in this study are contained within this article and supplementary materials.

## Supplementary Materials

Supplementary materials can be found at https://www.mdpi.com/1422-0067/22/2/928/s1.
