# Supplementary material for: Enhancing the Physiochemical Properties of Puerarin via L-Proline Co-Crystallization: Synthesis, Characterization, and Dissolution Studies of Two Phases of Pharmaceutical Co-Crystals
Source: Int J Mol Sci. 2021 Jan 18;22(2):928. doi: 10.3390/ijms22020928 (PMC7832312; doi:10.3390/ijms22020928)
Supplement: Supplementary file 1 [file ijms-22-00928-s001.pdf]

Supplementary Information for

Enhancing the Physiochemical Properties of Puerarin via L-Proline Co-crystallization: Synthesis, Characterization, and Dissolution Studies of Two Phases of Pharmaceutical Co-crystals

Muhammad Inam, Lu Liu, Jian-Wei Wang, Ka-Xi Yu, Chi-Uyen Phan, Jie Shen, Wen-Hua Zhang,

Guping Tang and Xiurong Hu*

Table of Contents

Figure S1 The ^{1}H NMR spectra of free PUE (a), PRO (b), **I** (c), and **II** (d). 3

Figure S2 The FT-IR spectra of **I** (a) and **II** (b). 4

Figure S3 The TGA-DSC diagrams of **I** (a) and **II** (b). 5

Figure S4. The cell viability data of PUE, **I**, and **II** against colon cancer CT-26 cell lines upon culturing 48 h. 6

Table S1 Hydrogen-bonding tables (Å and °) for **I** and **II**. 7

Table S2 A list of the solubility (mg/mL) of PUE, **I**, and **II**. 8

Table S3 Time-dependent dissolution rate (mg/mL) of PUE, **I**, and **II** in different medium. 9

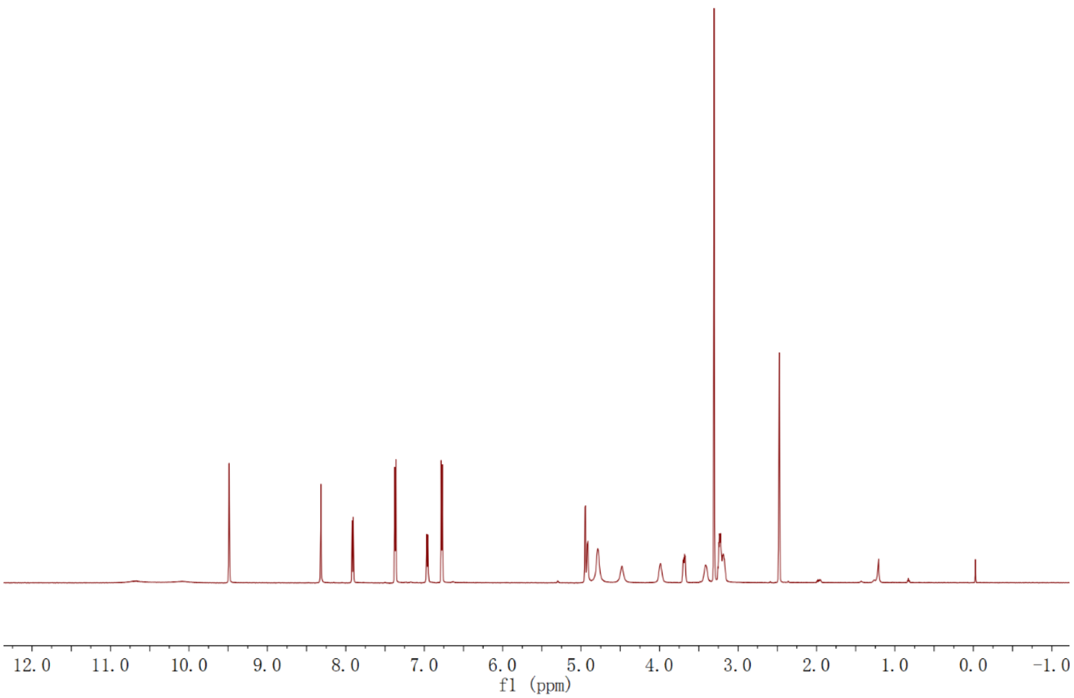
12.0 11.0 10.0 9.0 8.0 7.0 6.0 5.0 4.0 3.0 2.0 1.0 0.0 -1.0
f1 (ppm)

(a)

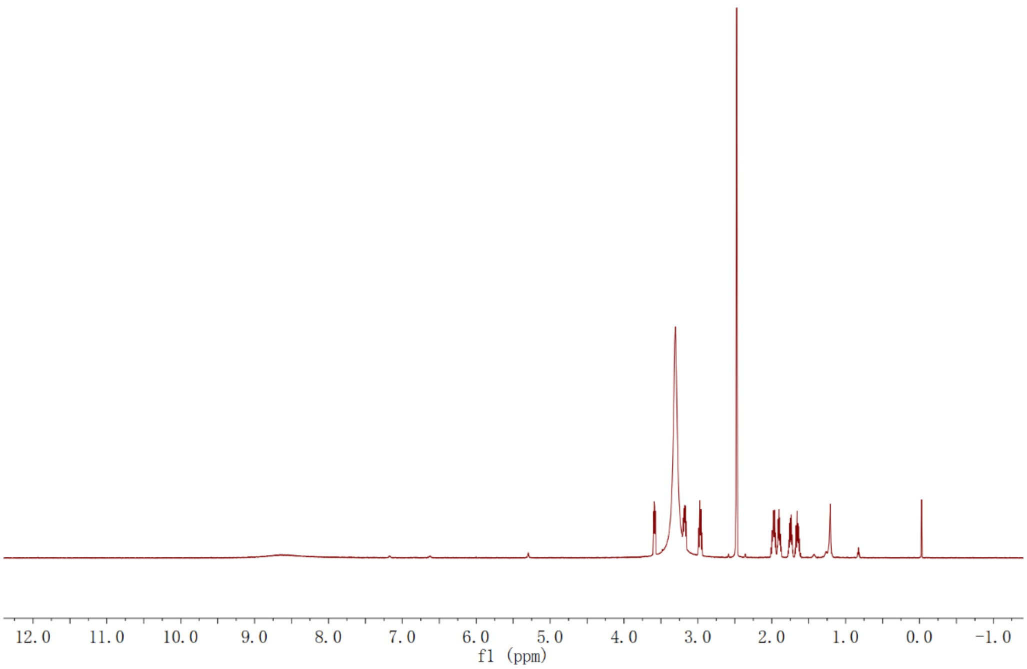
12.0 11.0 10.0 9.0 8.0 7.0 6.0 5.0 4.0 3.0 2.0 1.0 0.0 -1.0
f1 (ppm)

(b)

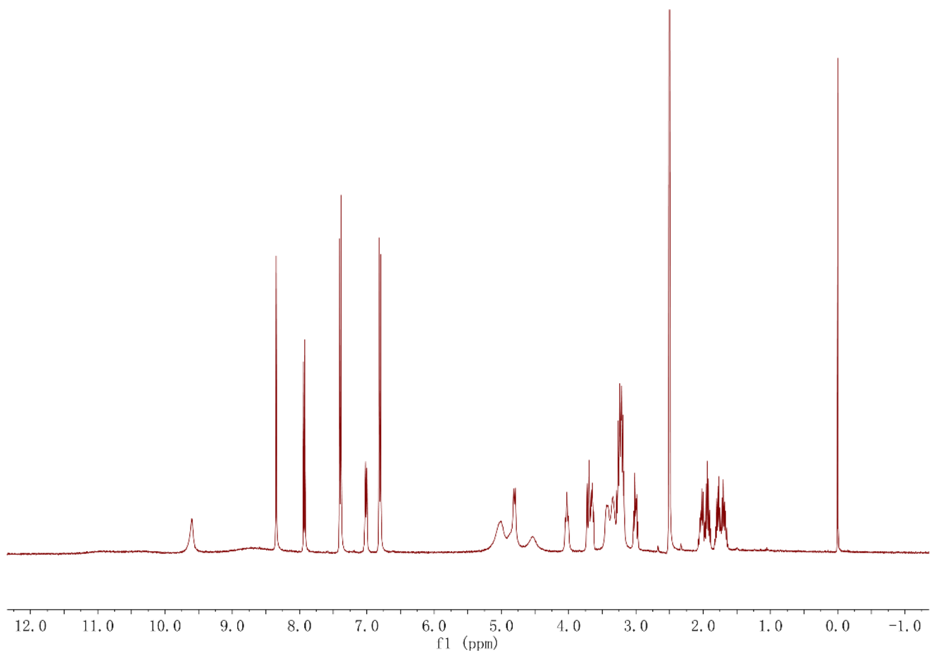

(c)

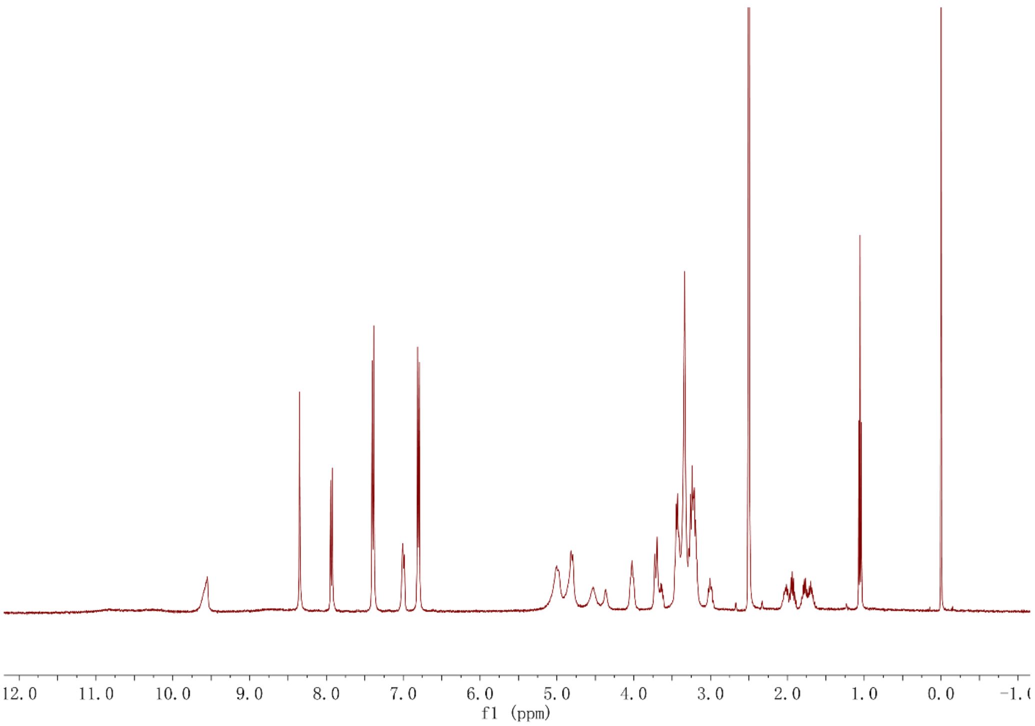

(d)

Figure S1 The ^{1}H NMR spectra of free PUE (a), PRO (b), **I** (c), and **II** (d).

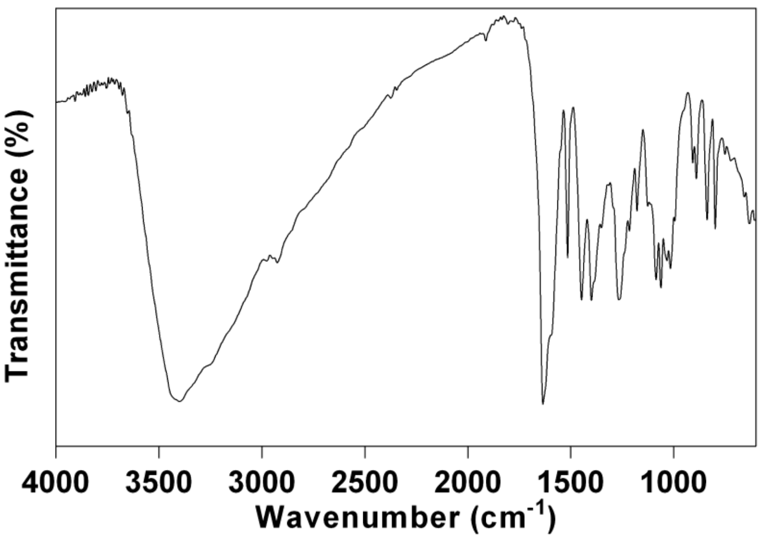

(a)

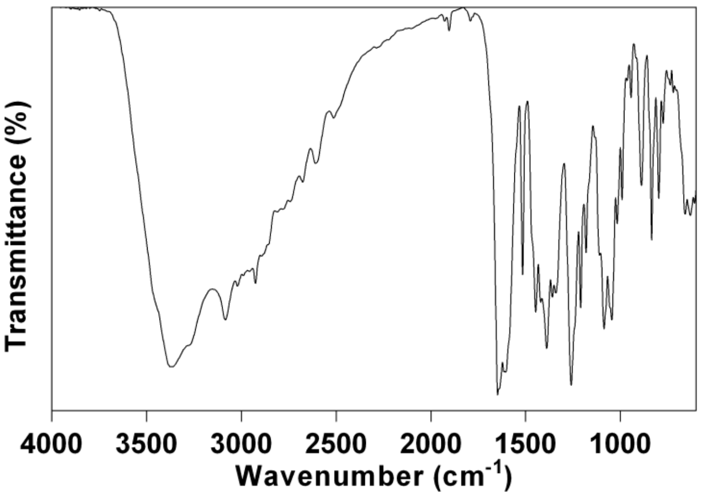

(b)

Figure S2 The FT-IR spectra of **I** (a) and **II** (b).

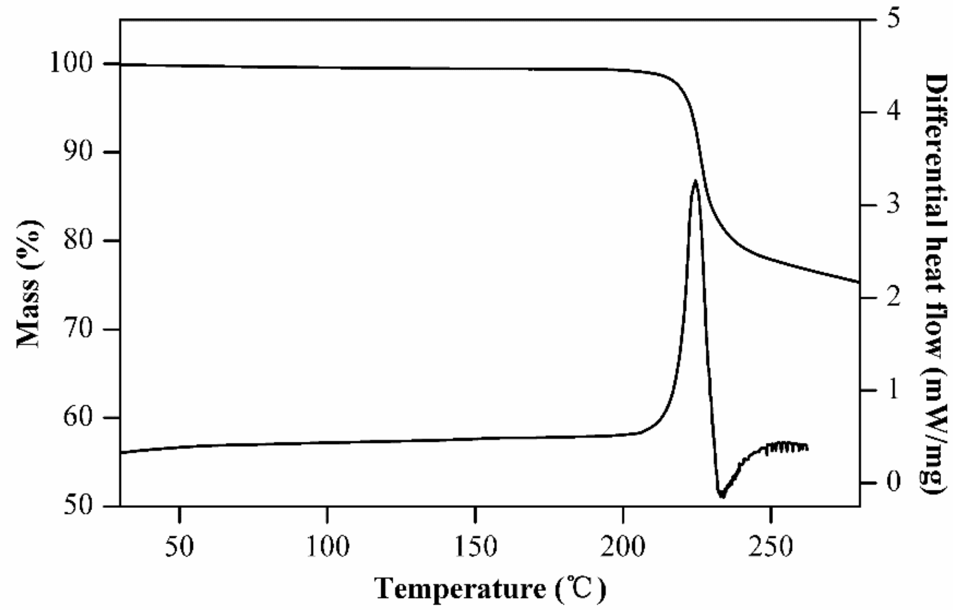

(a)

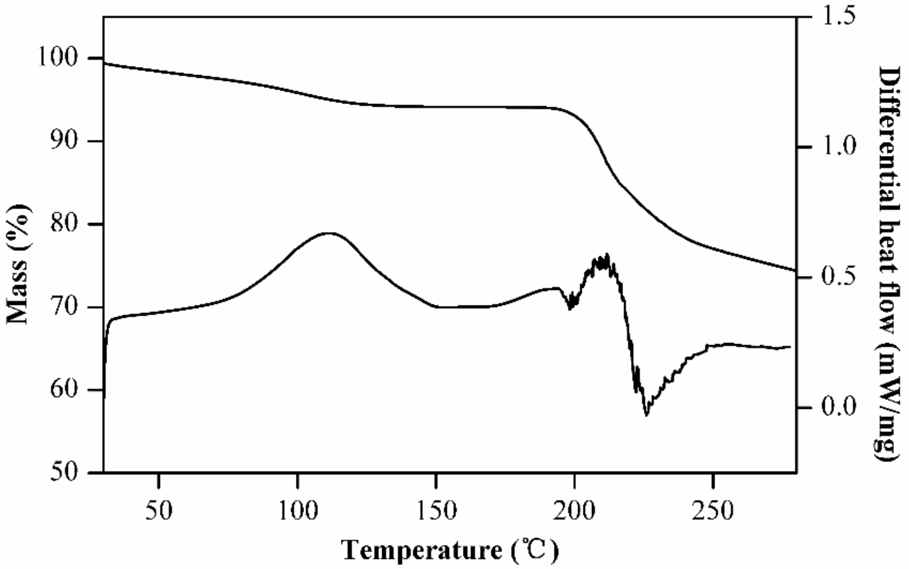

(b)

Figure S3 The TGA-DSC diagrams of **I** (a) and **II** (b).

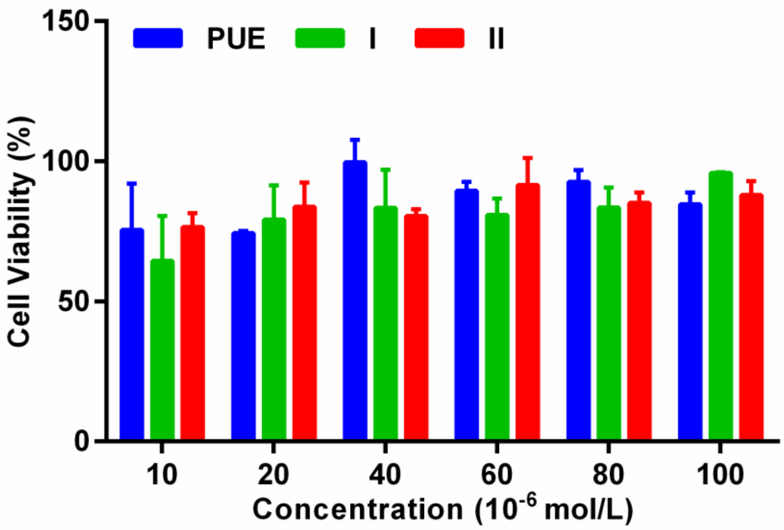

Figure S4. The cell viability data of PUE, **I**, and **II** against colon cancer CT-26 cell lines upon culturing 48 h.

Table S1 Hydrogen-bonding tables (Å and °) for **I** and **II**.

D-H···A	d(D-H)	d(H···A)	d(D···A)	∠(DHA)
[PUE][PRO] (**I**)				
O(7)-H(7)...O(8)#1	0.84	1.96	2.785(2)	168.3
O(9)-H(9)...O(6)#2	0.84	2.11	2.851(2)	146.3
O(9)-H(9)...O(1)#3	0.84	2.53	2.999(2)	116.5
O(8)-H(8)...O(10)	0.84	1.97	2.785(2)	164.0
O(6)-H(6)...O(3)#4	0.84	1.88	2.683(2)	160.1
O(4)-H(4)...O(5)	0.84	1.92	2.627(2)	141.8
O(1)-H(1)...O(11)#5	0.84	1.86	2.681(2)	164.5
N(1)-H(1B)...O(7)#6	0.91	2.10	2.886(2)	144.0
N(1)-H(1A)...O(10)#6	0.91	1.92	2.754(2)	151.8
N(1)-H(1B)...O(11)	0.91	2.19	2.661(2)	111.6
Symmetry codes: #1 −*x*, *y* − 1/2, −*z* + 2; #2 *x*, *y* + 1, *z*; #3 −*x* + 1, *y* + 1/2, −*z* + 1; #4 −*x*, *y* − 1/2, −*z* + 1; #5 *x*, *y*, *z*−1; #6 −*x* + 1, *y* + 1/2, −*z* + 2.				
$[PUE]_2[PRO]\cdot EtOH\cdot(H_2O)_2$ (**II**)				
O(1B)-H(1O1)...O(13)#1	0.83	2.09	2.819(3)	145.9
O(1A)-H(1A)...O(5B)#2	0.82	2.28	3.092(3)	172.6
O(1A)-H(1A)...O(6B)#2	0.82	2.41	2.862(3)	116.1
O(4A)-H(4A)...O(5A)	0.82	1.93	2.632(2)	143.7
O(4B)-H(1O4)...O(5B)	0.83	2.45	3.086(2)	133.7
O(6B)-H(6B)...O(8A)#3	0.82	1.94	2.756(3)	175.6
O(7B)-H(7B)...O(13)#4	0.82	1.95	2.770(2)	174.5
O(7A)-H(7A)...O(7B)#5	0.82	1.95	2.747(2)	165.1
O(8A)-H(8A)...O(10)	0.82	1.87	2.677(3)	165.9
O(8B)-H(8B)...O(10)#4	0.82	1.92	2.727(3)	167.8
O(14)-H(1W)...O(6B)#6	0.83	2.25	2.938(3)	140.4
O(14)-H(1W)...O(7A)#2	0.83	2.26	2.848(3)	128.5
O(14)-H(2W)...O(1A)#7	0.83	2.47	3.155(4)	139.9
N(1)-H(1C)...O(14)	0.89	1.91	2.786(3)	169.4
N(1)-H(1D)...O(11)	0.89	2.13	2.604(3)	112.5
N(1)-H(1D)...O(12)	0.89	2.05	2.841(4)	148.3
Symmetry codes: #1 *x* − 1, *y* + 1, *z*; #2 *x*, *y* − 1, *z*; #3 *x*, *y*, *z* + 1; #4 *x* − 1, *y*, *z* + 1; #5 *x* + 1, *y* ,*z* – 1; #6 *x*, *y* − 1, *z* − 1; #7 *x*, *y*, *z* − 1.				

Table S2 A list of the solubility (mg/mL) of PUE, **I**, and **II**.

Dissolution Medium	PUE		**I**		**II**	
	Average	SD (%)	Average	SD (%)	Average	SD (%)
Distilled Water	2.528	1.621	5.858	0.478	5.865	0.374
gastrointestinal tract at pH 1.2	1.513	1.247	3.972	0.249	3.470	1.674
phosphate buffer at pH 6.8	2.487	1.247	4.430	0.245	4.832	0.283

Table S3 Time-dependent dissolution rate (mg/mL) of PUE, **I**, and **II** in different medium.

	PUE		**I**		**II**	
	Average	SD (%)	Average	SD (%)	Average	SD (%)
Time (min)			**Water**			
5	0.062	0.000	0.220	0.094	0.131	0.094
10	0.109	0.094	0.343	0.125	0.210	0.082
15	0.190	0.125	0.452	0.047	0.281	0.170
20	0.250	0.327	0.533	0.294	0.457	0.125
25	0.294	0.386	0.591	0.205	0.567	0.047
30	0.506	0.047	0.674	0.189	0.701	0.125
40	0.611	0.216	0.767	0.163	1.450	0.082
50	0.692	0.356	0.945	0.327	2.110	0.082
60	0.821	0.170	1.045	0.368	2.490	0.000
80	0.896	0.082	1.373	0.330	2.840	0.000
100	0.935	0.125	1.503	0.283	3.203	0.424
120	1.031	0.170	1.645	0.400	3.410	0.818
			**pH 1.2**			
5	0.008	0.025	0.013	0.170	0.171	0.526
10	0.019	0.017	0.035	0.125	0.240	0.047
15	0.023	0.021	0.122	0.170	0.337	0.017
20	0.030	0.065	0.192	0.125	0.417	0.094
25	0.035	0.019	0.249	0.170	0.479	0.009
30	0.043	0.248	0.356	0.125	0.534	0.330
40	0.163	0.429	0.588	0.170	1.005	0.000
50	0.354	0.005	0.711	0.170	1.342	0.330
60	0.394	0.640	0.958	0.082	1.489	0.170
80	0.435	0.682	1.159	0.294	1.683	0.309
100	0.568	0.680	1.383	0.163	1.923	0.094
120	0.685	0.896	1.603	0.294	2.005	0.063
			**pH 6.8**			
5	0.078	0.000	0.207	0.047	0.121	0.170
10	0.110	0.205	0.291	0.082	0.212	0.125
15	0.168	0.125	0.384	0.094	0.352	0.170
20	0.244	0.125	0.493	0.125	0.446	0.189
25	0.274	0.094	0.570	0.082	0.537	0.082
30	0.347	0.082	0.664	0.141	0.861	0.047
40	0.415	0.082	0.786	0.170	1.059	0.047
50	0.444	0.082	0.850	0.047	1.503	0.471
60	0.504	0.094	0.918	0.205	2.210	0.047
80	0.560	0.000	1.183	0.170	2.723	0.250
100	0.641	0.082	1.265	0.082	3.138	0.589
120	0.785	0.050	1.303	0.170	3.370	0.245
